# A hydroponic approach to assess the morpho-physiological responses of cotton cultivars under varying vapor pressure deficit conditions

**DOI:** 10.3389/fpls.2026.1751642

**Published:** 2026-02-16

**Authors:** Kripa Dhakal, Magdalena Maria Julkowska, Avat Shekoofa

**Affiliations:** 1 Department of Plant Sciences, University of Tennessee, Knoxville, TN, United States; 2 FedEx Institute of Technology, University of Memphis, Memphis, TN, United States; 3 School of Integrative Plant Science, Plant Biology Section, Cornell College of Agriculture and Life Sciences, Ithaca, NY, United States

**Keywords:** cotton cultivars, hydroponic system, stomatal conductance, transpiration rate, vapor pressure deficit (VPD, kPa)

## Abstract

High temperature and water-deficit stresses are among the major limiting factors in cotton (*Gossypium hirsutum* L.) production. Increasing atmospheric vapor pressure deficit (VPD, kPa) due to global warming further challenges crop productivity by altering plant water balance. This study aimed to assess the effects of different VPD conditions on whole-plant growth characteristics and physiological parameters of new cotton cultivars grown in a hydroponic system. The experiment was conducted from December 2022 to April 2023 at the West Tennessee Research and Education Center (WTREC), University of Tennessee, Jackson, TN. Two Conviron chambers were maintained at two VPD levels (chamber 1, 1.1 kPa and chamber 2, 2.1 kPa, both at 32 °C). Four cotton cultivars DP 2131 B3TXF (Thryvon technology), PHY 400 W3FE, PHY 1140A385–04 W3FE, and PHY 1140B373–04 W3FE were transplanted into hydroponic containers at the cotyledon stage. Cultivar DP 2131 B3TXF exhibited the highest plant height, root length, and total fresh and dry weight in both chambers compared to other cultivars. Under high VPD (2.1 kPa), DP 2131 B3TXF had significantly higher transpiration rate (TR) and stomatal conductance (gs) compared to PHY 400 W3FE. Interestingly, PHY 400 W3FE demonstrated higher intrinsic water use efficiency (iWUE) and significantly lower TR than DP 2131 B3TXF at the same VPD level. The same pattern was observed among cultivars for their stomatal density in chamber 2. These findings indicate that these new cotton cultivars performed differently when comparing TR, stomatal density, and root growth parameters under high VPD conditions. PHY 400 W3FE expressed the limited TR trait, while DP 2131 B3TXF exhibited greater biomass production and root length compared to other cultivars despite reduced root elongation under high VPD conditions.

## Introduction

Cotton (*Gossypium hirsutum* L.) is a major fiber crop increasingly exposed to climate related stresses including heat and limited water availability, which threaten its productivity and long-term sustainability. Cotton is highly responsive to its environment, particularly vapor pressure deficit (VPD), understanding cultivar-specific responses to different VPD levels is essential for improving water use efficiency, growth, and yield under changing climatic conditions. Additionally, increasing atmospheric VPD due to global warming poses a critical challenge by influencing the water balance of the plants (Carmo-Silva et al., 2012; Grossiord et al., 2020; Franco-Navarro et al., 2025). VPD is a key environmental parameter used to measure atmospheric water demand by combining the effects of air temperature and relative humidity (Broughton and Conaty, 2022; Shekoofa et al., 2023). A high VPD indicates a hot and dry condition, while a low VPD indicates a cooler and more humid environment (Shekoofa et al., 2016; Sinclair et al., 2017). It has been identified that high VPD (kPa) rates influence plant physiological responses, particularly transpiration, water uptake, and stomatal conductance in crops including cotton (Devi and Reddy, 2018; Broughton et al., 2021; Shekoofa et al., 2021; Wedegaertner et al., 2022). Under high VPD, plants are forced to increases their transpiration rate. When this demand exceeds plants could respond by partially closing stomata to conserve water. Although this response helps maintain plant water status, it reduces stomatal conductance and photosynthesis, creating a trade-off between water conservation and growth (Sinclair and Ludlow, 1986; Reddy et al., 2005).

While previous studies have demonstrated that cotton exhibits distinct physiological and morphological responses to high VPD conditions, the variability of these responses among different cultivars remains unclear. For instance, plants respond to changes in VPD between the leaf and the atmosphere through changes in stomatal activities (Devi and Reddy, 2018; Shekoofa et al., 2021). Increasing VPD has been shown to linearly increase transpiration rate at the leaf level (Rawson et al., 1977; Yong et al., 1997; Devi and Reddy, 2018; Shekoofa et al., 2021), despite a decrease in stomatal conductance (g_s_), which has been observed in leaves of glasshouse-grown cotton (Duursma et al., 2013).

Studies have also shown that g_s_ decreases with increasing VPD, although the precise mechanism is not clear (Yong et al., 1997; Conaty et al., 2014); in most cases, g_s_ decreases exponentially with increasing VPD. Still, there may be differences in physiological responses between cotton cultivars (Devi and Reddy, 2018; Shekoofa et al., 2021). There is also evidence that increasing VPD rates can cause inhibition of photosynthesis unrelated to stomatal closure (Pettigrew et al., 1990). Drought and temperature stresses usually result in reduced air humidity; thus, it is possible that besides soil water availability and temperature, the relative air humidity also plays a role in the growth of plants under stress (Sinclair et al., 2017; Grossiord et al., 2020).

Utilizing a hydroponic system allows for precise and controlled assessment of cotton cultivars, offering valuable insights into their growth and developmental characteristics. Furthermore, hydroponics serves as a robust assessment tool for early detection of contrasting phenotypes, providing a reliable platform for screening plants based on their morphological and physiological responses (Onanuga and Adl, 2012). This study aimed to investigate the morpho-physiological responses of four cotton cultivars to different VPD levels in a hydroponic setting. The research focused on determining the effects of low and high VPD conditions on key physiological parameters such as stomatal conductance (g_s_), transpiration rate (TR), and intrinsic water use efficiency (iWUE) to identify cultivars with potential resilience to climate-induced stress.

## Materials and methods

This study was conducted from December 2022 to April 2023 at the West Tennessee Research and Education Center (WTREC), University of Tennessee, Jackson, TN. Two Conviron growth chambers (Conviron MTR30, Winnipeg, Manitoba, CA) were used to maintain different VPD conditions through the life of experiment [chamber 1: (1.1) kPa and chamber 2: (2.1) kPa, both at a constant temperature of 32 °C)]. Air temperature and relative humidity were recorded every 5 min using a humidity/temperature digital data logger (Lascar Electronics, Erie, PA). Light intensity in both chambers ranged from 500 to 550 μmolm^-2^s^-1^.

Four cotton cultivars were selected based on their diverse genetic backgrounds, technological traits, and previously observed transpiration responses under high VPD and temperature stress (Shekoofa et al., 2021; Wedegaertner et al., 2023). The cultivars included ‘Phytogen (PHY) 400 W3FE’, ‘PHY 1140A385–04 W3FE’, ‘PHY 1140B373–04 W3FE’ (Phytogen Cottonseed, Corteva Agroscience, Indianapolis, IN), ‘DeltaPine (DP) 2131 B3TXF’ (Deltapine Cottonseed, Bayer CropScience, St. Louis, MO) (with ThryvOn technology). The uniform-sized seeds of each cultivar were selected. Before sowing, the seeds were surface-sterilized by dipping them in a bleach and water solution (3:1) for 2–3 minutes, followed by thorough washing with distilled water (Kelly et al., 2023). Seeds were then sown in plastic flat trays filled with potting mix and placed inside an incubator set to 30–32°C with 75% relative humidity and 16:8-hrs. (light: dark) photoperiod.

The hydroponic system was constructed using 20-gallon plastic containers fitted with PVC pipe and sprayers (**Supplementary Figure S1**). A motor pump (400 GPH Active Aqua Pump) continuously circulated the nutrient solution throughout the system. When the seedlings reached the 2–3 true leaf stage, seedlings were gently washed to remove potting media and transferred into the net cups (7.6 cm) positioned in the hydroponic lids. Containers were filled with 57 L of nutrient solution [Flora Micro (N-P-K: 5-0-1) 1.8 mL L^-1^, Flora Gro (N-P-K: 2-1-6) 1.7 mL L^-1^, and Flora bloom (N-P-K: 0-5-4) 1.26 mL L^-1^] and placed inside the growth chambers (**Supplementary Figure S2**). The experiment was arranged in a randomized complete block design, with four replicates per cultivar in each VPD treatment. Replications 1 & 2 were conducted from December 8, 2022, to February 7, 2023, and replications 3 & 4 from February, 2023 to April 25, 2023. Replications were staggered due to chamber availability, but environmental conditions and procedures were identical across all replications.

### Data collection

#### Growth and total plant biomass

Plant height was measured from the base to the tip of the highest point of the plant using a meter stick. Root length was recorded by measuring the longest root, from the root crown (the point where the roots meet the stem) to the tip of the longest root. A ruler was used to measure the root length. Plant height and root length were measured weekly.

At the experiment termination (50 days after planting), the total shoot fresh biomass was recorded using a digital scale (± 0.1 g). To determine dry biomass, the fresh plant material (i.e., above-ground plant biomass and root) was placed in paper bags and dried in an oven at 60°C for 40 hours. After drying, the plant material was weighed again to obtain the dry biomass.

#### Stomatal microscopy

The youngest fully expanded leaf was selected from each plant for their stomatal analysis. A thin layer of clear nail polish base/topcoat (Beauty 21 Cosmetics, Ontario, CA) was applied to both adaxial and abaxial leaf surfaces and allowed to dry for 5 min. A strip of transparent tape (19 mm diameter, 3 M, St. Paul, MN), approximately 6 cm in length was pressed onto the dried nail polish, which was then peeled off with the tape. The tape with the nail polish imprint was immediately affixed to a glass slide for microscopy observations (Purdom et al., 2022; Li et al., 2025). Two samples were collected from each plant, one from the abaxial (underside) and one from the adaxial (upper side) leaf surfaces. The samples were examined using a Nikon Eclipse E600 microscope (Tokyo, Japan) equipped with an MU300 digital camera (AmScope, Irvine, CA) at 20 × magnification. A 1 cm^2^ area where the nail polish leaf imprint was most intact was selected for analysis. Using AmScope imaging software, a series of non-overlapping images (0.50 × 0.45 mm) was captured in a grid pattern within the selected zone (Li et al., 2025). Stomatal density (SD) was calculated by averaging the number of stomata in ten images from each sample (Purdom et al., 2022).

#### Physiological parameters

Stomatal conductance (g_s_, mol H_2_O m^-2^ s^-1^) and photosynthetic rate (A, μmol CO_2_ m^-2^ s^-1^) were measured individually for each plant from each chamber using a portable photosynthesis system Li-Cor 6400-XT (Li-Cor Inc. Biosciences, Lincoln, NE). A 6 cm^2^ section of leaf was enclosed in the instrument chamber and allowed to equilibrate to the chamber’s environmental condition for 60 seconds before a measurement was taken. The measurements were taken weekly. The leaf section within the Li-Cor 6400 chamber was exposed to 32 °C and 1000 μmol m^-2^s^-1^ photosynthetically active radiation (PAR) using a 6400–01 light source. The flow rate was 500 μmol s^-1^, and the CO_2_ concentration was maintained at 400 μmol CO_2_ mol^-1^ air. Intrinsic water use efficiency (iWUE, μmol CO_2_ mol^-1^ H_2_O) was calculated using Equations (1) & (2) by including, VPD (kPa), stomatal conductance (g_s_, mol H_2_O m^-2^ s^-1^), photosynthesis (A, μmol CO_2_ m^-2^ s^-1^), and transpiration rate (TR, mmol m^-2^ s^-1^):

(1) $$TR=VPD\times g_{s}$$

(2) iWUE=P/TR

#### Root morphology

At the end of the experiment, root samples (one per cultivar per replication) were collected from each cultivar to assess root morphological traits. A 2-cm segment was excised from the primary root of each plant. These root samples were preserved in 70% ethanol and sent to Boyce Thompson Institute for detailed root morphology analysis. The analysis focused on several key traits related to root structure and function, including total root area, xylem area, xylem vessel area, and the number of xylem vessels. The roots were hand-sectioned, stained with toluidine blue O solution (0.25% w/v) for 3 minutes, and subsequently washed with distilled water to remove excess of stain.

The cross sections of individual roots were imaged over multiple snapshots using Diaphot TMD Inverted Microscope (Nikon). The individual images depicting one root were subsequently combined into one picture using Adobe Photoshop Photomerge feature. All sections were subsequently quantified using ImageJ, and the individual features were visualized and compared for their respective sizes using R software (full pipeline available at https://rpubs.com/mjulkowska/Cotton_root_Avat2023).

### Data analysis

All data collected were analyzed using JMP Pro 17 (SAS Institute Inc., Cary, NC). Data were first checked for normality and homogeneity of variance. A two-way analysis of variance (ANOVA) was used with VPD levels and cultivars as fixed effects. When significant effects were detected treatment means were separated using Tukey’s Honest Significant Difference (HSD) test with statistical significance set at *p* < 0.05. Significant interaction effects were further examined by comparing cultivar’s responses within each VPD level to interpret how VPD influenced each cultivar’s growth and physiological traits.

## Results

### Morphological parameters

#### Plant height

**Figure 1:**
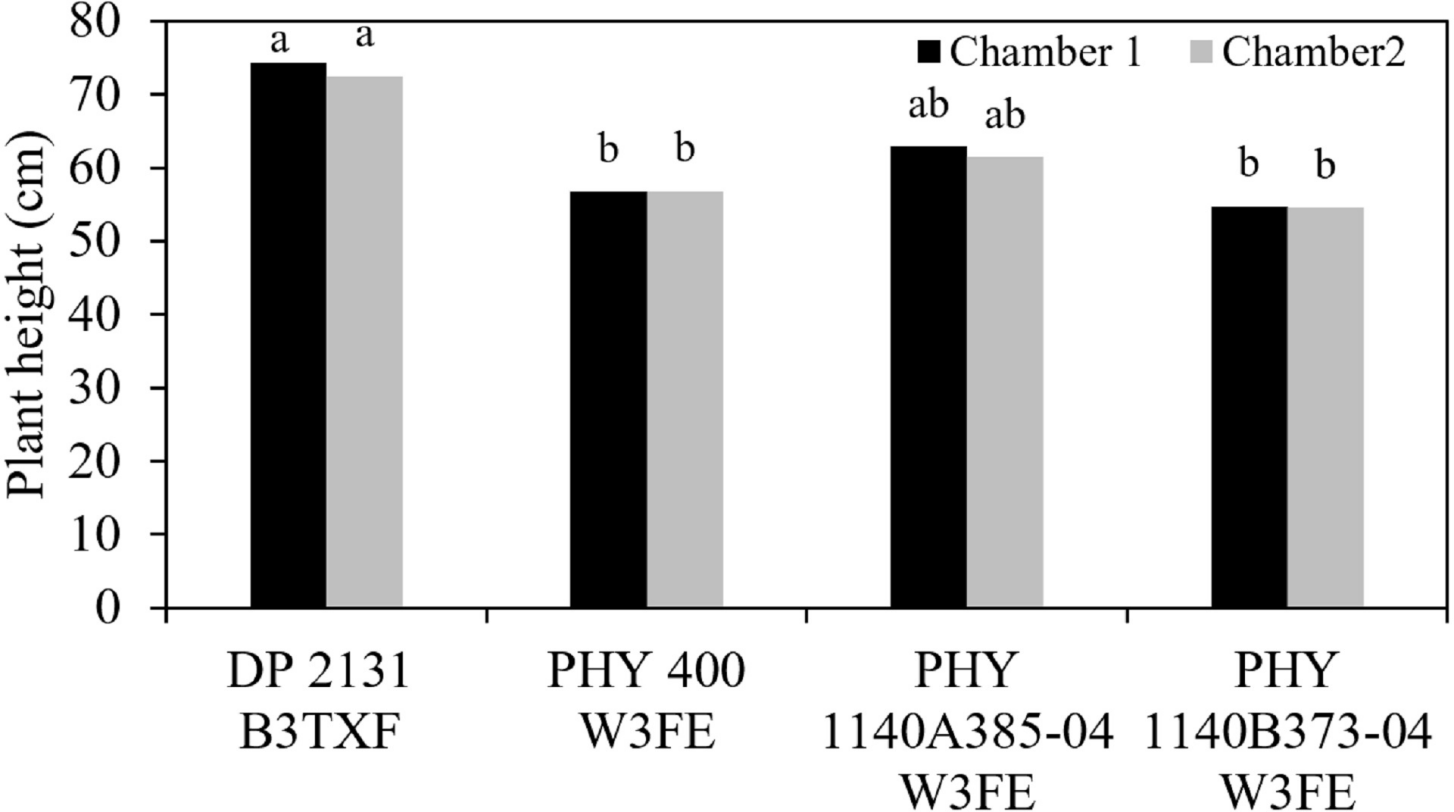
Cotton cultivars mean plant height under two different environmental conditions, chamber 1: VPD 1.1 kPa, chamber 2: VPD 2.1 kPa. Different lowercase letters in the column denote significant differences at *P<* 0.05.

Plant height measurements across four cultivars grown under two distinct VPD conditions revealed significant cultivar-specific responses to atmospheric water demand (**Figure 1**). Cultivar DP 2131 B3TXF had the highest plant height in both chambers (74.30 and 72.49cm, respectively), under different VPD conditions (chamber 1: 1.1 kPa and chamber 2: 2.1 kPa, both at 32°C) compared to the other cultivars. In contrast, PHY 400 W3FE and PHY 1140B373–04 W3FE had significantly shorter plant heights in chamber 1, with similar values observed in chamber 2. Cultivar PHY 1140A385–04 W3FE showed intermediate plant heights, measuring 62.87 cm in chamber 1 and 61.42 cm in chamber 2, with no significant difference from either cultivar, the tallest (DP 2131 B3TXF) and shortest cultivars.

#### Root length

**Figure 2:**
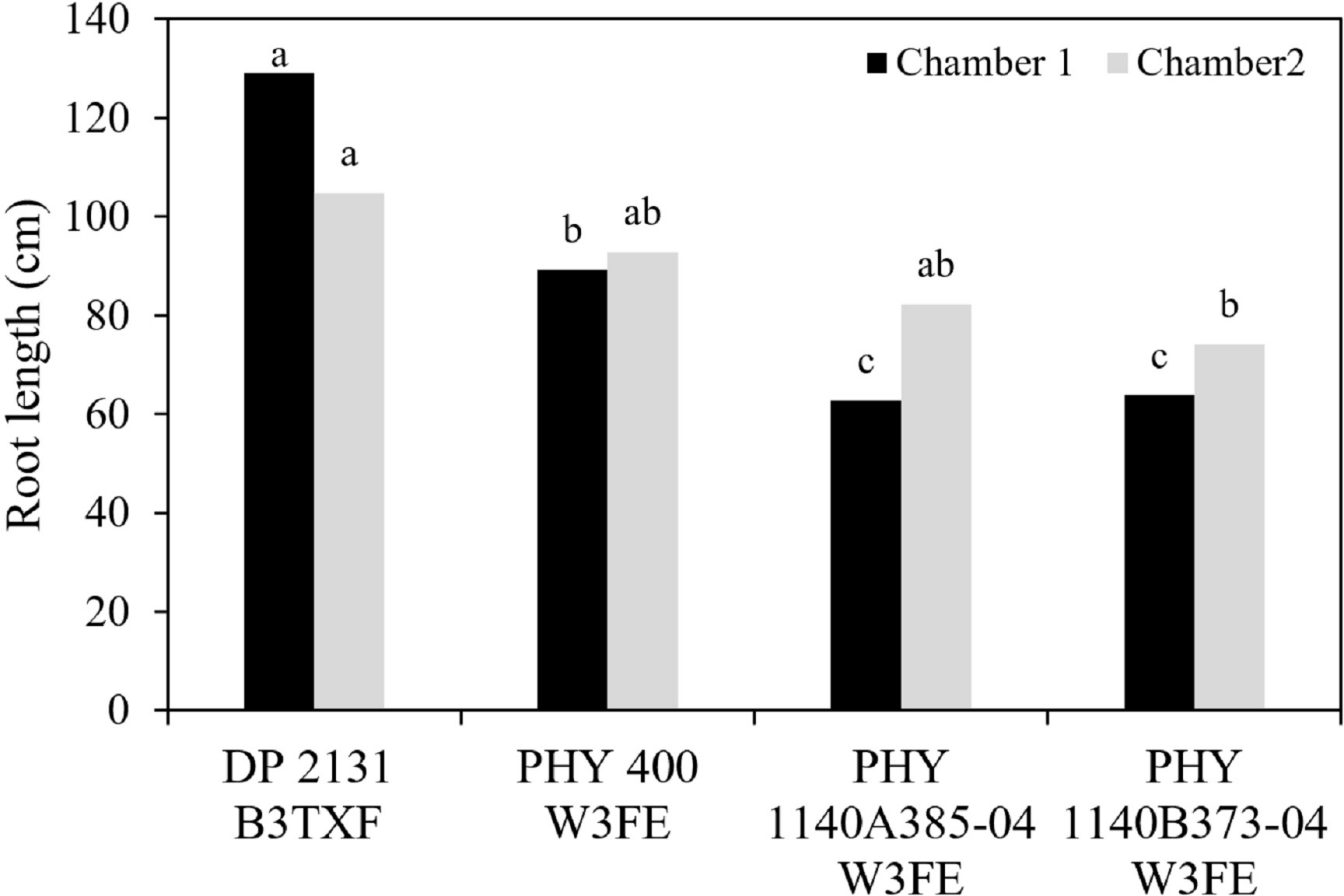
Mean root length of four cotton cultivars growing in two different environmental conditions chamber 1: VPD 1.1 kPa, chamber 2: VPD 2.1 kPa. Different lowercase letters in the column denote significant differences at *P<* 0.05.

The experiment demonstrated variation in root length across cultivars and chambers (**Figure 2**). Cultivar DP 2131 B3TXF exhibited the longest root length in both chambers compared to the rest of cultivars (**Figure 2**). PHY 400 W3FE had an intermediate root length in chamber 1 (i.e., under 1.1 kPa, VPD), while in chamber 2 (i.e., under 2.1 kPa, VPD), its root length was not significantly different from DP 2131 B3TXF. Cultivars PHY 1140A385–04 W3FE and PHY 1140B373–04 W3FE had the shortest root lengths in chamber 1, but both showed increased in their root lengths in chamber 2 (**Figure 2**). The significant decrease in root length for DP 2131 B3TXF between the two chambers (from 129.16 cm to 104.83 cm) suggests that chamber 2, with the lower relative humidity and high VPD rate, may provide a less favorable condition for the cultivar’s optimal root development. In contrast, cultivars including PHY 400 W3FE, PHY 1140A385–04 W3FE, and PHY 1140B373–04 W3FE showed less variation and even had longer root growth in chamber 2 (**Figure 2**).

#### Fresh and dry weight

**Figure 3:**
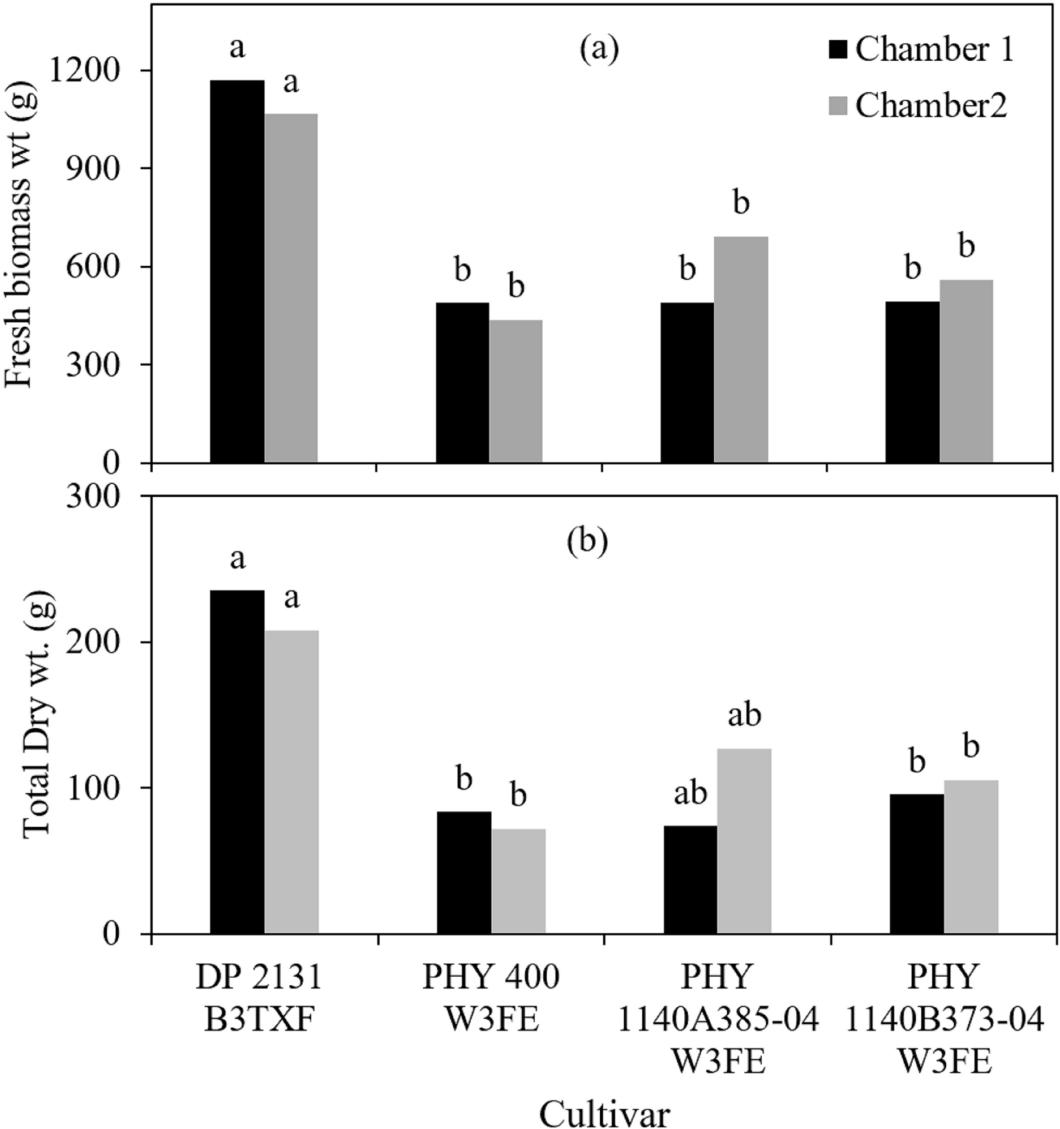
**(A)** Fresh plant biomass, **(B)** total dry weight of four cotton cultivars 50 days after planting (at the end of experiment/termination) growing in two different environmental conditions chamber 1: VPD 1.1 kPa, chamber 2: VPD 2.1 kPa. Different lowercase letters in the column denote significant differences at *P<* 0.05.

The fresh and dry biomass data revealed significant cultivar-specific responses to varying VPD rates (**Figures 3A, B**). Across both chambers, cultivar DP 2131 B3TXF (1170 g in chamber 1: VPD 1.1 kPa, and 1065.75 g in chamber 2: VPD 2.1 kPa) consistently had the highest fresh plant biomass. **Figure 3A** illustrates that the remaining cultivars PHY 400 W3FE, PHY 1140A385–04 W3FE, and PHY 1140B373–04 W3FE had significantly lower fresh biomass compared to DP 2131 B3TXF, but they were not significantly different from each other in both chambers. Similar to the fresh weight, cultivar DP 2131 B3TXF exhibited the highest dry weight in both chambers (235.25 g in chamber 1 and 208 g in chamber 2). In contrast, cultivar PHY 400 W3FE had the lowest dry weight in both chambers (**Figure 3B**). Additionally, the dry weight of cultivar PHY 1140A385–04 W3FE was not statistically different from either the highest or lowest values across both chambers.

#### Root morphology

**Figure 4:**
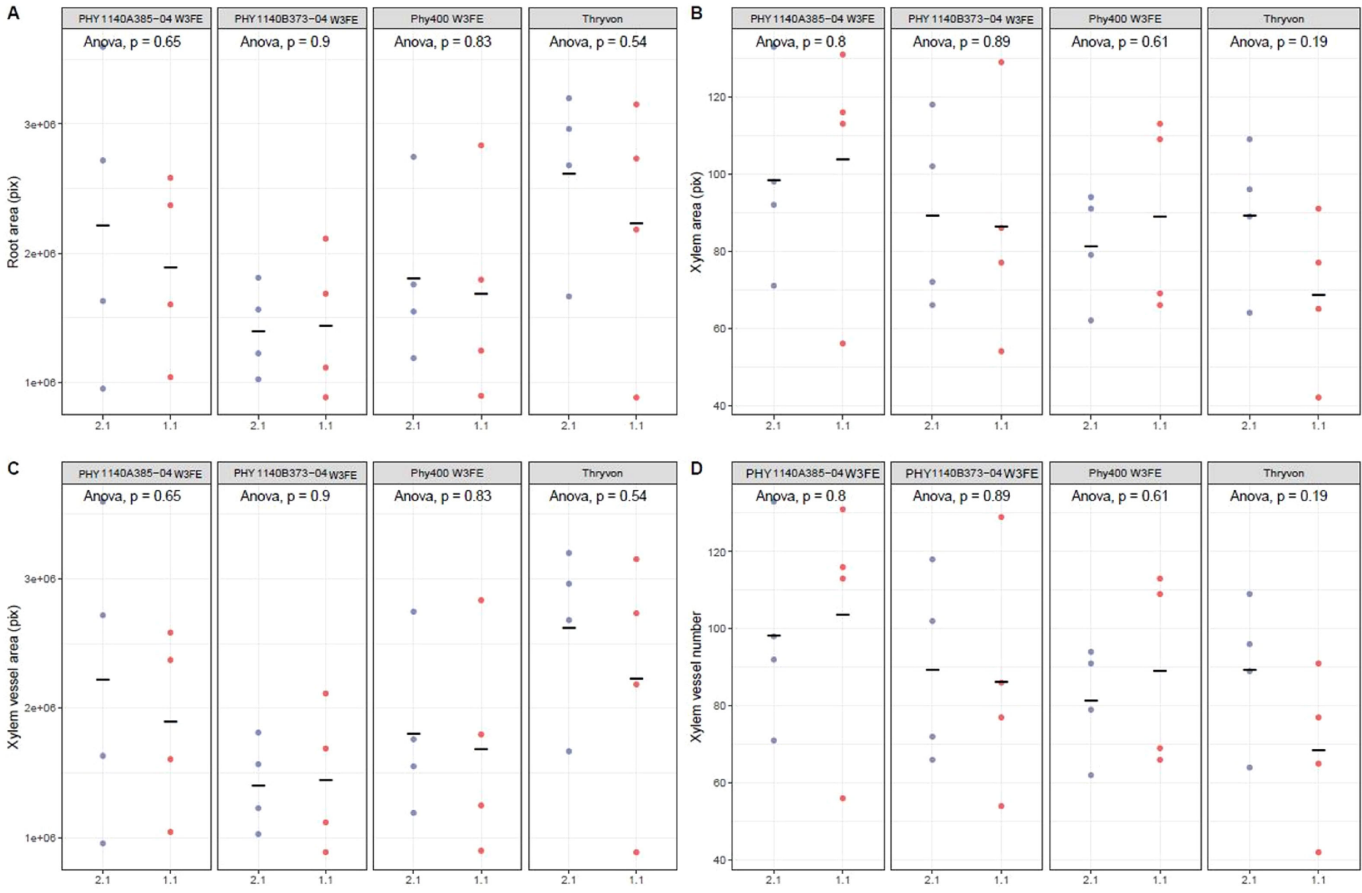
**(A)** Root area, **(B)** Xylem area, **(C)** Xylem vessel area and **(D)** Xylem vessel number of four cotton cultivars 50 days after planting (at the end of experiment/termination) growing in two different environmental conditions chamber 1: VPD 1.1 kPa, chamber 2: VPD 2.1 kPa.

The anatomical and morphological traits of cotton roots measured under different VPD conditions showed no statistically significant differences among the four cultivars (**Figure 4**). However, numerical trends suggested cultivar-specific responses. Cultivars PHY 1140A385–04 W3FE and PHY 400 W3FE showed slight increases in root area, xylem area, and xylem vessel area under high VPD (2.1 kPa). Although these differences were not statistically significant, they may indicate a tendency toward increased hydraulic capacity under elevated evaporative demand. In contrast, DP 2131 B3TXF showed relatively stable or slightly reduced values across all root morphological parameters under high VPD.

### Physiological parameters

**Figure 5:**
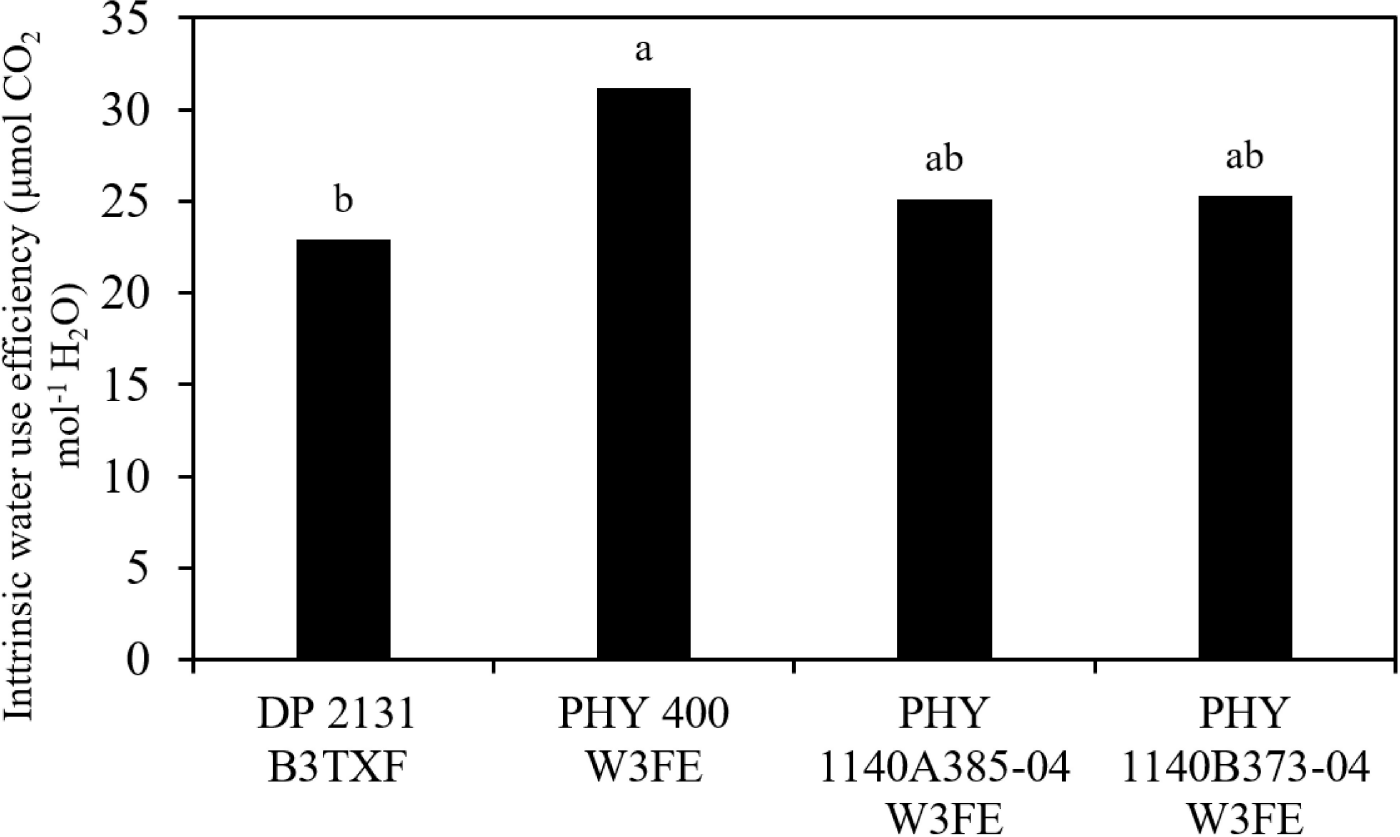
Cotton cultivars’ mean intrinsic water use efficiency (iWUE, μmol CO_2_ mol^-1^ H_2_O) at chamber 2: VPD 2.1 kPa. Different lowercase letters in the column denote significant differences at *P<* 0.05.

Significant differences in transpiration rate (TR, mmol m^-2^ s^-1^), photosynthetic rate (A, μmol CO_2_ m^-2^ s^-1^), stomatal conductance (g_s_, mol H_2_O m^-2^ s^-1^), and intrinsic water use efficiency (iWUE) values were observed among the four tested cultivars in chamber 2 (**Table 1**). These physiological parameters were significantly higher for DP 2131 B3TXF compared to PHY 400 W3FE under high VPD (chamber 2: 2.1 kPa), indicating higher gas exchange and growth potential, but at the cost of higher water use (**Table 1**). In contrast, PHY 400 W3FE had the lowest values for all parameters, suggesting the more efficient use of available water specifically by reducing photosynthetic activities. Cultivars PHY 1140A385–04 W3FE and PHY 1140B373–04 W3FE demonstrated intermediate values across all parameters. Interestingly, PHY 400 W3FE had significantly lower TR compared to DP 2131 B3TXF in chamber 2 (VPD, 2.1 kPa).We observed that cultivar PHY 400 W3FE had significantly higher intrinsic water use efficiency (iWUE) than other cultivars in chamber 2 (**Figure 5**). There were no significant differences in iWUE and other parameters for DP 2131 B3TXF compared to PHY 400 W3FE at chamber 1 with VPD 1.1 kPa (**Table 1**).

**Table 1:** Mean transpiration rate, photosynthesis, stomatal conductance, and intrinsic water use efficiency of four cotton cultivars grown in chamber 1 (VPD 1.1 kPa) and chamber 2 (VPD 2.1 kPa).

Cultivars		Transpiration rate (TR, mmol m^-2^ s^-1^)	Photosynthesis (A, μmol CO_2_ m^-2^ s^-1^)	Stomatal conductance (g_s_, mol H_2_O m^-2^ s^-1^)	Intrinsic water use efficiency (iWUE, μmol CO_2_ mol^-1^ H_2_O)
Chamber 1	DP 2131 B3TXF	0.11	11.96 a	0.10	22.13
	PHY 400 W3FE	0.13	11.63 a	0.11	21.29
	PHY 1140A385–04 W3FE	0.11	10.88 ab	0.09	19.48
	PHY 1140B373–04 W3FE	0.11	9.55 b	0.10	16.69
	*P-value*	NS	0.0004	NS	NS
Chamber 2	DP 2131 B3TXF	0.25 a	14.23 a	0.11 a	22.9 b
	PHY 400 W3FE	0.14 b	11.27 b	0.06 b	31.14 a
	PHY 1140A385–04 W3FE	0.21 ab	12.65 ab	0.10 a	25.12 ab
	PHY 1140B373–04 W3FE	0.19 ab	12.76 ab	0.08 ab	25.29 ab
	*P-value*	0.013	0.0008	0.0017	0.0368

Different lowercase letters in the column denote significant differences at P< 0.05.

NS, Non-significant at P< 0.05.

#### Stomatal density

**Figure 6:**
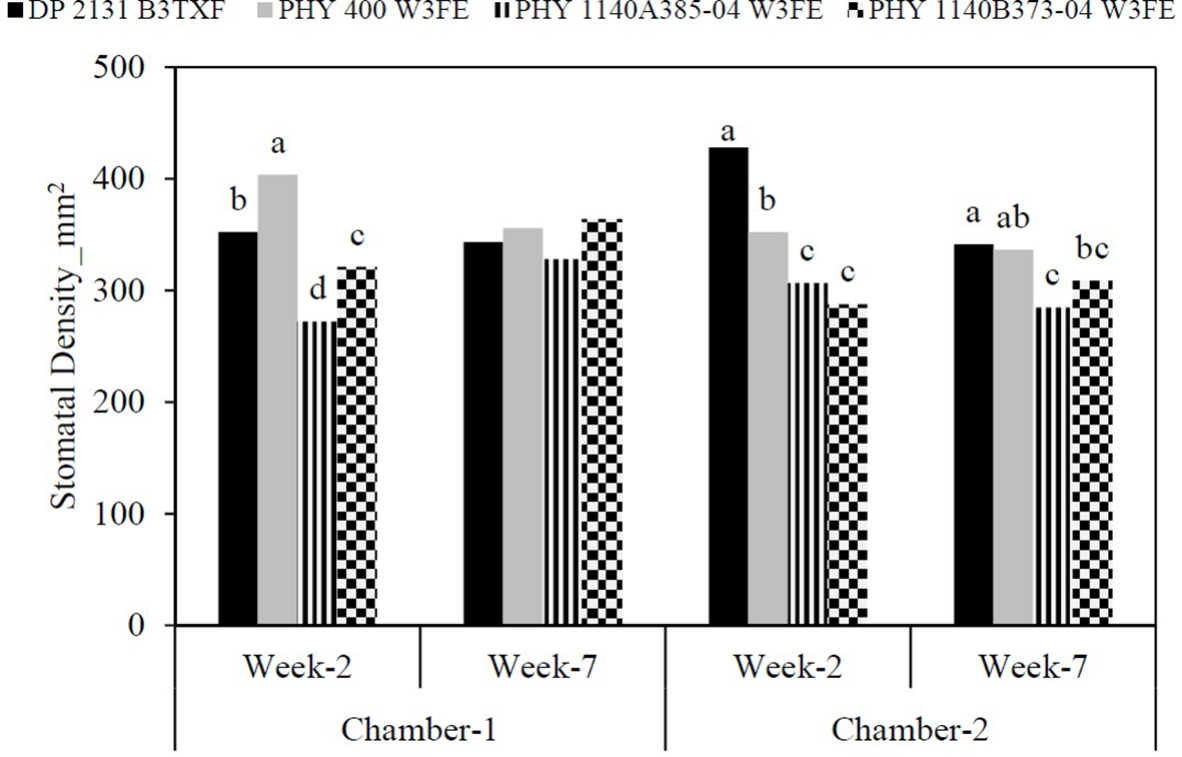
Stomatal density of four cotton cultivars in two different environmental conditions measured in week-2 and week-7 after transplanting in a hydroponic solution. Different lowercase letters in the column denote significant differences at *P<* 0.05.

Stomatal density was measured at two different time points: Week 2 and Week 7 after transplanting into the hydroponic system, corresponding to the early and later stages of vegetative growth. The data showed significant differences among the cultivars (**Figure 6**). Under chamber 1, PHY 400 W3FE exhibited the highest stomatal density at Week 2 (403.75 mm^-2^), followed by DP 2131 B3TXF (352.49 mm^-2^), PHY 1140B373–04 W3FE (320.97 mm^-2^), and PHY 1140A385–04 W3FE (271.94 mm^-2^). By Week 7, only two cultivars, PHY 1140A385–04 W3FE and PHY 1140B373–04 W3FE, showed an increase in stomatal density reaching 328.06 mm^-2^ and 363.47 mm^-2^, respectively.

In contrast, in chamber 2, DP 2131 B3TXF had the highest stomatal density at Week 2 (428.05 mm^-2^), suggesting a strong initial developmental response to high VPD condition. However, this cultivar showed a marked decline by Week 7 (341.25 mm^-2^). PHY 400 W3FE and PHY 1140A385–04 W3FE exhibited reductions in stomatal density from Week 2 to Week 7, except for PHY 1140B373–04 W3FE, which slightly increased (from 287.63 to 308.61 mm^-2^) (**Figure 6**).

## Discussion

This study provides a controlled assessment of cultivar-specific differences in root anatomy, stomatal density, and water-use efficiency in cotton under varying VPD conditions. Using a hydroponic system maintained at a constant temperature at two VPD levels, we observed significant variation in morphological and physiological traits among cultivars. The cultivars differed not only in growth characteristics such as plant height and root length, but also in transpiration rate, stomatal regulation, and intrinsic water-use efficiency. Plant height and root length, which are key indicators of early vigor and stress tolerance (Mahmood et al., 2022), were significantly affected by VPD levels, highlighting the impact of atmospheric evaporative demand on cultivar performance.

Our results demonstrated that cultivars differed in both growth and water use strategies. DP 2131 B3TXF consistently exhibited the greatest plant height and root length under both VPD conditions, suggesting strong growth potential under both low and high VPD. In contrast, PHY 400 W3FE, PHY 1140A385–04 W3FE, and PHY 1140B373–04 W3FE demonstrated more stable plant and root growth under higher VPD conditions. This pattern reflects a water saving strategy based on partial stomatal closure, even at the expense of photosynthesis. These patterns align with observations by Devi and Reddy (2018) and Shekoofa et al. (2021), they reported that some cotton cultivars possess a limited-transpiration trait under high VPD, enabling them to conserve water while maintaining core physiological functions. This suggests a potential adaptive mechanism that allow these cultivars to maintain development despite elevated atmospheric evaporative demand. Each cultivar’s distinct genetic makeup influences its growth characteristics, including root development. For example, DP 2131 B3TXF may possess superior genetic traits that promote longer root growth in both chambers compared to other cultivars.

The numerical trends observed in root morphological traits under varying VPD conditions suggest cultivar-specific anatomical responses, despite the lack of statistical significance. Notably, PHY 1140A385–04 W3FE and PHY 400 W3FE had slight numerical increases in root area, xylem area, and xylem vessel area under high VPD (2.1 kPa). Although these differences were not statistically significant, they may suggest a trend toward enhanced root hydraulic capacity, consistent with patterns reported from the study (Vadez, 2014). In contrast, DP 2131 B3TXF maintained relatively stable root anatomical traits across VPD treatments, suggesting a more conservative strategy that may rely on physiological regulation rather than structural changes, which is consistent with a prior study that some cotton cultivars regulate transpiration primarily through stomatal or hormonal adjustments (Devi and Reddy, 2018; Grossiord et al., 2020; Franco-Navarro et al., 2025). These findings provide new evidence that cotton cultivars employ distinct combinations of structural and physiological strategies to cope with atmospheric water demand. Physiological measurements further highlighted the cultivar-specific nature of water-use strategies. PHY 400 W3FE adopted a water-conserving strategy, with significantly lower g_s_, TR, and P, particularly under high VPD. This supports the concept of partial stomatal closure to restrict transpirational water loss, even at the cost of reduced photosynthesis (Pettigrew et al., 1990). Muchow and Sinclair (1989) also confirmed that low stomatal density was associated with low epidermal conductance in sorghum genotypes. The higher iWUE of PHY 400 W3FE in chamber 2 indicates efficient use of available water and potential advantage under water-limited conditions (Shekoofa et al., 2021; Wedegaertner et al., 2022). Similar strategies have been reported in other crops such as peanut and sorghum, where reduced stomatal aperture under high VPD improved drought resilience (Devi et al., 2010; Shekoofa et al., 2014). Similarly, in maize, Shekoofa et al. (2016) and Sinclair et al. (2017) reported that hybrids with higher iWUE and lower stomatal conductance under high evaporative demand conditions maintained greater yields under stress. Together these findings support the value of limited transpiration and high iWUE as selection traits for stress resilience across crop species.

Stomata play a crucial role in balancing photosynthetic carbon fixation and water loss and strongly influence plant water use efficiency. Previous studies have demonstrated that cotton stomatal conductance exhibit high sensitivity to variation in VPD rates (Devi and Reddy, 2018; Shekoofa et al., 2021; Wedegaertner et al., 2022; 2023). In our study, stomatal density varied among cultivars between the early (week 2) and later (week 7) vegetative growth stages under both VPD conditions. These results indicated the cultivar-specific regulation of stomatal development in response to VPD rates. Notably, PHY 1140B373–04 W3FE demonstrated greater plasticity in stomatal density over time under high VPD. Although stomatal density decreased for most cultivars between Week 2 and Week 7, relative differences between cultivars remained, with DP 2131 B3TXF and PHY 400 W3FE continuing to exhibit higher stomatal densities. Cultivars exhibiting reduced stomatal density and stronger stomatal regulation under high VPD generally demonstrated lower transpiration rates while maintaining greater iWUE. Few studies have confirmed that plants exposed to long-term high VPD changes in stomatal density and size (Fanourakis et al., 2013), and the stomatal length and width increased, but its density decreased (Jiao et al., 2022).

The hydroponic setting eliminated soil-related variability, allowing a more accurate assessment of cultivar responses to atmospheric conditions. Temperature was held constant at 32 °C, allowing the isolated effect of VPD to be assessed. While VPD is a crucial atmospheric factor, it rarely acts in isolation. In field conditions, VPD interacts with fluctuating temperature, light, soil moisture, and rooting constraints, all of which influence transpiration, photosynthesis, and hormone signaling (Grossiord et al., 2020). Therefore, cultivars identified here as water-saving or growth-oriented under controlled conditions should be validated across diverse environments and seasons to confirm their field performance stability. Controlled-environment studies like this remain valuable for identifying stress-resilient cultivars, but their predictions must be interpreted cautiously when extrapolated to field conditions.

## Conclusion

Cotton cultivars had distinct strategies to cope with high VPD, balancing growth, water conservation, and root development in this study. Differences in stomatal density and regulation directly influenced transpiration and intrinsic water-use efficiency, highlighting key physiological traits that can be targeted in breeding programs. The results can guide breeders in selecting cultivars with traits that enhance water-use efficiency and resilience under elevated evaporative demand. The controlled hydroponic system provided a reliable platform for identifying these physiological differences at an early developmental stage, independent of soil variability. However, field conditions expose crops to interacting stresses such as fluctuating temperature, soil water deficits, and variable rooting depth, which may intensify or alter their morpho-physiological responses to VPD levels. Therefore, future research should be considered under field conditions to confirm cultivars performance across variable environments and ensure cultivars performance in water-limited cotton production areas.

## Data Availability

The original contributions presented in the study are included in the article/**Supplementary Material**, further inquiries can be directed to the corresponding author/s.

## Supplementary material

Supplementary Figure 1 The structure of each hydroponic container, piping frame, and a cotton cultivar transferred into a net cup.

Supplementary Figure 2 Cotton cultivars transferred into hydroponic containers and placed inside each growth chamber.
