# Supplementary material for: A hydroponic approach to assess the morpho-physiological responses of cotton cultivars under varying vapor pressure deficit conditions
Source: Front Plant Sci. 2026 Feb 16;17:1751642. doi: 10.3389/fpls.2026.1751642 (PMC12950770; doi:10.3389/fpls.2026.1751642)
Supplement: Supplementary Figure 1 — The structure of each hydroponic container, piping frame, and a cotton cultivar transferred into a net cup. [file DataSheet1.docx]

**Supplementary file**

**Figure 1S.** The structure of each hydroponic container, piping frame, and a cotton cultivar transferred into a net cup.


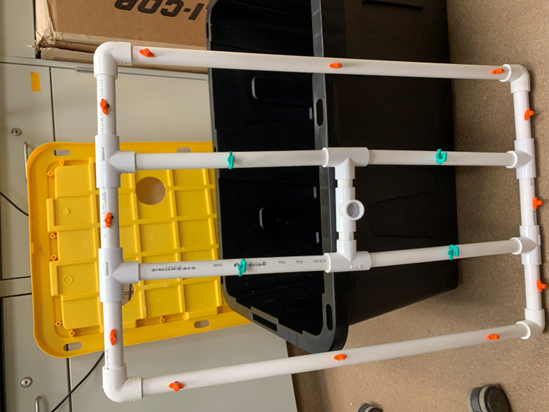

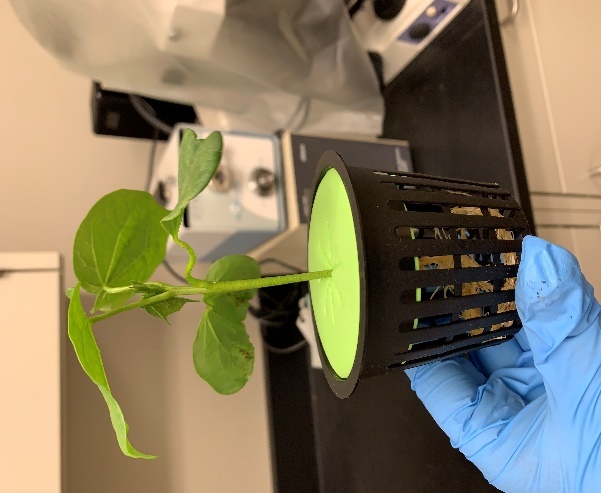

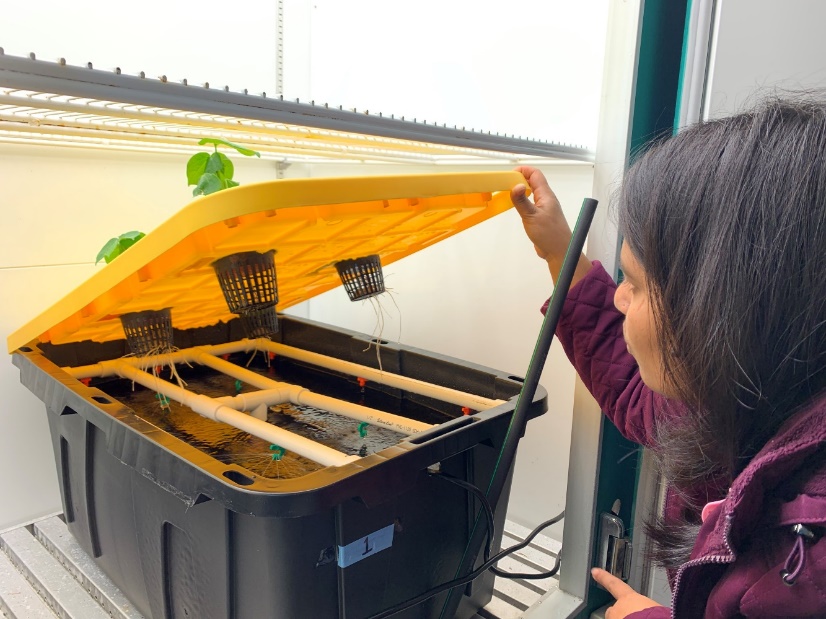

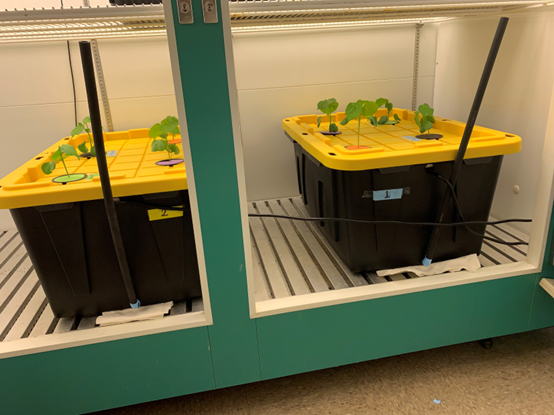


**Figure 2S.** Cotton cultivars transferred into hydroponic containers and placed inside each growth chamber.
